# Erratum: Debons et al. Magnetic Field Alignment, a Perspective in the Engineering of Collagen-Silica Composite Biomaterials. *Biomolecules* 2021, *11*, 749

**DOI:** 10.3390/biom11081117

**Published:** 2021-07-29

**Authors:** Nicolas Debons, Kenta Matsumoto, Noriyuki Hirota, Thibaud Coradin, Toshiyuki Ikoma, Carole Aimé

**Affiliations:** 1 Laboratoire de Chimie de la Matière Condensée de Paris (LCMCP), Sorbonne Université, CNRS, 75005 Paris, France; debons.nicolas@hotmail.fr (N.D.); thibaud.coradin@sorbonne-universite.fr (T.C.); 2 Tokyo Institute of Technology, School of Materials and Chemical Technology, Department of Materials Science and Engineering, Ookayama 2-12-1, Meguro-ku, Tokyo 152-8550, Japan; matsumoto.k.be@m.titech.ac.jp (K.M.); tikoma@ceram.titech.ac.jp (T.I.); 3 National Institute for Materials Science, Fine Particles Engineering Group, 3-13 Sakura, Tuskuba 305-0003, Japan; hirota.noriyuki@nims.go.jp; 4 Ecole Normale Supérieure, CNRS-ENS-SU UMR 8640, 24 rue Lhomond, 75005 Paris, France

In the original article, there was a mistake published in Figure 2. The figure was published twice in the original published version [1]. The corrected Figure 2 is shown below. We apologize for any inconvenience caused, and state that the scientific conclusions are unaffected. The original article has been updated.

## Figures and Tables

**Figure 2:**
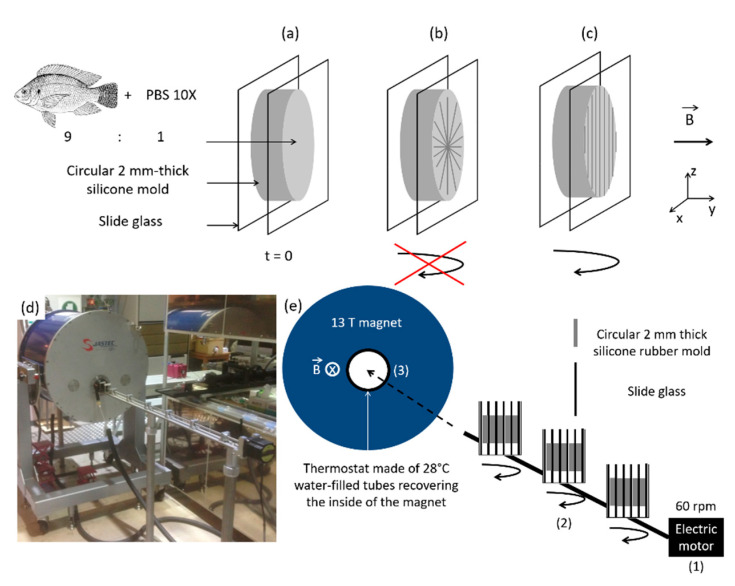
(**a**) Magnetic field application in a collagen gel. (**b**) Under a high magnetic field along y axis and without rotation, collagen fibers perpendicularly align against magnetic field (xz plan). (**c**) Upon rotation, only fibrils along the axis of rotation (z axis) remain. (**d**) Photo of the set-up, and (**e**) scheme of the essential characteristics: electric motor for stirring samples, magnetic field and thermostat.
