# Supplementary material for: Magnetic Field Alignment, a Perspective in the Engineering of Collagen-Silica Composite Biomaterials
Source: Biomolecules. 2021 May 18;11(5):749. doi: 10.3390/biom11050749 (PMC8157240; doi:10.3390/biom11050749)
Supplement: Supplementary file 1 [file biomolecules-11-00749-s001.zip › biomolecules-1203544-supplementary.pdf]

Magnetic field alignment, a perspective in the engineering of collagen-silica composite biomaterials

Nicolas Debons [1], Kenta Matsumoto [2], Noriyuki Hirota [3], Thibaud Coradin [1], Toshiyuki Ikoma [2] and Carole Aimé [1,4*]

1 Sorbonne Université, CNRS, Laboratoire de Chimie de la Matière Condensée de Paris (LCMCP), Paris, F-75005, France
2 Tokyo Institute of Technology, School of Materials and Chemical Technology, Department of Materials Science and Engineering, Ookayama 2-12-1, Meguro-ku, Tokyo 152-8550, Japan.
3 National Institute for Materials Science, Fine Particles Engineering Group, 3-13 Sakura, Tuskuba 305-0003, Japan.
4 Ecole Normale Supérieure, CNRS-ENS-SU UMR 8640, 24 rue Lhomond, Paris, 75005, France
* Correspondence: carole.aime@ens.psl.eu

Supporting Information

Polarized light microscopy (PLM) was used for its sensitivity to the birefringence of materials reflecting aligned structures. Regarding pure collagen membranes, before EDC treatment (Figure S1a1,a2), the contrast in terms of brightness between two cross polarizers was less important than after EDC treatment (Figure S1b1,b2). This indicates that the cross-linking favored the alignment of the fibrils.

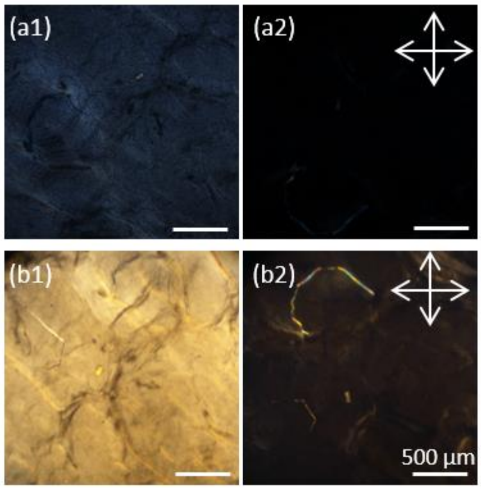

Figure S1 PLM images of pure collagen films (a) before and (b) after EDC crosslinking. PLM imaging was performed by taking 2 serial images with the sample oriented *vs* the cross polarizers (white arrows) at maximal transmission (1) and at extinction (2).
